# “When I am breathless now, I don’t have the fear that’s linked to it”: a case series on the potential of EMDR to break the dyspnea-anxiety cycle in COPD

**DOI:** 10.1186/s12890-022-02250-1

**Published:** 2022-12-01

**Authors:** Kris Mooren, Kirsten Smit, Yvonne Engels, Daisy Janssen, Judith Godschalx

**Affiliations:** 1 Department of Pulmonology, Spaarne Gasthuis, Boerhaavelaan 22, 2035 RC Haarlem, The Netherlands; 2 Department of Anesthesiology, Pain and Palliative Medicine, Radboud University Medical Center, Nijmegen, The Netherlands; 3 Department of Health Services Research and Department of Family Medicine, Care and Public Health Research Institute, Faculty of Health Medicine and Life Sciences, Maastricht University, Maastricht, The Netherlands; 4 Department of Psychiatry and Medical Psychology, Flevoziekenhuis, Almere, The Netherlands

**Keywords:** COPD, Breathlessness, EMDR

## Abstract

**Background:**

Expectations can enhance the intensity and the neural processing of breathlessness. Previous breathlessness episodes may influence the perception of subsequent episodes because of psycho-traumatic consequences. In post-traumatic stress disorder, eye movement desensitization and reprocessing (EMDR) is the therapy of choice.

**Aims and objectives:**

We explored the hypothesis that EMDR in patients with chronic obstructive pulmonary disease (COPD) and previous severe breathlessness episodes, improves breathlessness mastery by decreasing the anxiety component.

**Methods:**

As we found no literature on previous research on this subject, we undertook a qualitative case series on four patients with COPD GOLD 4/D and refractory breathlessness who wished to undergo EMDR for psychotraumatic breathlessness episodes. Amongst others, we used the Chronic Respiratory Disease Questionnaire (CRQ) before and after EMDR, and semi-structured, face-to-face, in-depth interviews.

**Results:**

All patients had between three and five EMDR sessions. On CRQ, subset mastery, three patients had a large improvement and one patient a moderate improvement. On subset emotional functioning, three patients showed a large improvement and one showed no change. All patients made a distinction between ‘regular’ breathlessness and breathlessness intertwined with anxiety. They all stated that the anxiety component of their breathlessness diminished or disappeared. All four would recommend EMDR for other COPD patients.

**Conclusion:**

There is ground for a randomized controlled clinical trial to test the effects of EMDR on breathlessness mastery in a subset of COPD patients with previous severe breathlessness episodes and high levels of anxiety.

**Supplementary Information:**

The online version contains supplementary material available at 10.1186/s12890-022-02250-1.

## Key message

This case series uses mixed methods to explore EMDR as a novel intervention in COPD patients with severe breathlessness. Our results support our hypothesis that EMDR may improve breathlessness mastery in patients with COPD and previous severe breathlessness crises, by decreasing the anxiety component.

**Supplementary Information:**

The online version contains supplementary material available at 10.1186/s12890-022-02250-1.

## Introduction

Chronic obstructive pulmonary disease (COPD) is an often progressive and life-threatening lung disease. It is the third leading cause of death in the world [1] and its prevalence is increasing worldwide. The cardinal symptom of COPD is breathlessness, which has a large impact on the quality of life in the majority of patients [2]. Breathlessness in COPD is known to correlate poorly with pulmonary function tests such as forced expiratory volume in the first second (FEV_1_) [3]. This discordance between physiological disturbances and the patient’s experience underscores the role of the brain in processing sensory signals from the respiratory system. Both mood and expectations are major contributors to the function of the brain networks that generate breathlessness perception [4]. Indeed, the brain generates predictions about the sensations the body should be feeling, often fed by prior experiences and contextual cues. For example, a staircase that has led to a breathlessness crisis before, may induce the same sensations in a patient before even starting the ascent [5].

As we have learned from patient experts, a breathlessness crisis is a paralyzing experience in which nothing is present except the terrible need to get air in [6, 7]. In a listing of dozens forms of torture, according to the ratings of perceived distress and uncontrollability, asphyxiation ranked first with respect to perceived uncontrollability and second with respect to perceived distress [8]. Of note, the defining characteristic of a psychotraumatic event is its capacity to provoke fear or helplessness in response to the threat of dying. It is likely that patients who experience breathlessness crises share characteristics with those who suffer from post-traumatic stress disorder (PTSD) [9]. Indeed, patients with recurrent COPD exacerbations have an increased risk of PTSD symptoms [10].

Unfortunately, in patients with severe COPD, distress not only is caused by airflow obstruction but may also contribute to it. Distress may lead to rapid, shallow breathing. This breathing pattern leads to air trapping, thus aggravating the breathlessness because of expiratory airway collapse [11]. Thus, the learning experience of a breathlessness crisis is susceptible for reinforcement, when a subsequent episode of breathing discomfort sets a “dyspnea-anxiety-dyspnea cycle” in motion.

Although our knowledge of the role of the brain in breathlessness perception has greatly increased in the last decade, this has not yet led to clinically applicable improvements for patients. In randomized controlled trials neither antidepressants nor anxiolytic drugs have been proven to alter the experience of patients with refractory breathlessness [12, 13]. However, the viewpoint that traumatic breathlessness episodes may lead to PTSD symptoms which aggravate breathlessness, has not been explored before.

The therapy of choice for PTSD, according to the World Health Organization (2013), is Eye Movement Desensitization and Reprocessing (EMDR). EMDR is a psychotherapeutic approach, developed over 30 years ago to treat traumatic memories and their associated stress symptoms [14]. Its effectiveness in PTSD has been proven in several meta-analyses [15, 16]. EMDR combines different well-established psychotherapeutic techniques such as imagined exposure, psychoeducation, cognitive change and self-control, with bilateral sensory stimulation (such as left-right eye movements or tapping on the knees) while the patient concentrates on the memory. According to the theory of adaptive information processing (AIP) that underpins EMDR, overwhelming psychotrauma causes physiological responses to interfere with information processing. As a consequence, a distressing event is stored in a raw, state-specific form. Activation of pathogenic memories can lead to symptoms such as flashbacks. Dysfunctionally stored memories are the focus of EMDR procedures. Supposedly, the vividness and emotionality of the pathogenic memory decreases during eye movements or other dual tasks, so that afterwards the memory can be reprocessed without the accessory stress and anxiety [17].

Patients with COPD are sporadically referred to undergo EMDR because of psychotraumatic events in their past. We hypothesize that EMDR in patients with COPD and previous severe breathlessness episodes improves breathlessness mastery by decreasing the anxiety component.

## Methods

A thorough search of the literature was performed on EMDR for COPD-related breathlessness (search terms and strategy described in Additional file 1). We found no previous research on this subject, although two case reports concerning the positive effect of EMDR on breathlessness due to other conditions—pediatric asthma and amyotrophic lateral sclerosis—were retrieved [18, 19].

Therefore, we undertook a qualitative case series on four patients with COPD GOLD 4/D and refractory breathlessness who wished to undergo EMDR for psychotraumatic breathlessness episodes. The case series employed mixed methods of quantitative outcome assessment and qualitative interviews. In our large teaching hospital, we consecutively included patients between December 2019 and March 2021, with the following inclusion criteria:

- COPD as diagnosed by a chest physician;
- Refractory severe breathlessness, defined as Modified Medical Research Council (mMRC) dyspnea scale 3–4, persisting despite optimal treatment;
- Referred by their chest physician for EMDR because of one or more stressful episodes in their past related to breathlessness;
- Willingness to be interviewed after the intervention.

Exclusion criteria were:

- Inability to read and write Dutch (for questionnaires);
- Inability to visit the hospital for appointments.

Patients were recruited from the pulmonary outpatient clinic.

The study protocol was approved by the Medical Ethics Review Committee of Amsterdam University, which confirmed that the Medical Research Involving Human Subjects Act (WMO) did not apply to the current study.

### Intervention

All patients were treated with the EMDR standard protocol by the same psychiatrist (JG), who has experience with administering EMDR since 2015 and has been an EMDR practitioner since 2019.

### Data collection

The following questionnaires were used before and directly after the intervention:

- *Clinical COPD Questionnaire (CCQ)* This is a simple 10-item, health-related quality of life questionnaire. It consists of three subdomains: symptoms, functional state and mental state. Items are scored on a Likert scale (range 0–60). The final score is the sum of all items divided by 10; separate scores for all three domains can be calculated. Higher scores indicate a worse health status. The minimal important difference of the CCQ total score is 0.4 [20].
- *Chronic Respiratory Disease Questionnaire (CRQ)* This questionnaire measures 4 domains, namely dyspnea, fatigue, emotional function and mastery, using a 7-point modified Likert Scale. Higher scores indicate better health-related quality of life. A change in the score of 0.5 on the 7-point scale, reflects a clinically significant small change. A change of 1.0 reflects a moderate change and a difference of 1.5 represents a large change [21].
- *Hospital Anxiety and Depression Scale (HADS)* HADS is a 14-item scale with seven items each for anxiety (HADS-A) and depression (HADS-D) subscales. Scoring for each item ranges from zero to three. A subscale score above eight denotes clinically relevant symptoms of anxiety or depression [22].
- *48-item Symptom Questionnaire (SQ48)* SQ48 is a self-report measure of psychological distress, using nine subscales (normal values between brackets) {range between curly brackets}: Depression (0–8){0–24}, Anxiety (0–11){0–24}, Somatization (0–8){0–28}, Agoraphobia (0–2){0–16}, Aggression (0–5){0–16}, Cognitive problems (0–11){0–20}, Social Phobia (0–9){0–20}, Work functioning (this item was not used since none of our patients were working), and Vitality (9–24){0–24} [23].

All patients reported their experience regarding psychotraumatic breathlessness experiences, their impact on their quality of life, and EMDR, in semi-structured, face-to-face, in-depth interviews. The interviews were all held after the EMDR, were audio-recorded and transcribed verbatim.

### Analysis

The scores of CRQ, CCQ, HADS and SQ48 were calculated before and after EMDR. Only descriptive statistics were used. Two researchers (KM and YE) independently open coded the first interview line by line with direct content analysis and compared and discussed the codes until consensus was reached. The remaining three interviews were coded by one researcher (KM). Next, two researchers (KM, YE) grouped the codes into themes and sub-themes. The coding process was performed using Atlas.ti software v.8 (http://atlasti.com; Atlas.ti Scientific Software Development GmbH, Berlin, Germany).

## Results

During the study period (between December 2019 and March 2021), nine patients with severe COPD, severe breathlessness and a wish to be referred for EMDR were assessed for inclusion. Of those, five were not included for the following reasons: one did not consent to be interviewed, two wanted EMDR for non-breathlessness related events, one declined from EMDR after the intake, and one died of an acute exacerbation of COPD (AECOPD) shortly after referral. The other four eligible patients were included in the case series. The most relevant scores of their questionnaires before and after EMDR are presented in Table 1.

**Table 1:** Outcomes of most relevant questionnaires before and after the intervention

	Case 1	Case 2	Case 3	Case 4
SQ, anxiety, before EMDR	12	16	16	20
SQ, anxiety, after EMDR	7*	14*	15*	8*
HADS-A, before EMDR	Missing	16	17	9
HADS-A, after EMDR	Missing	12*	11*	4*
CCQ, mental, before EMDR	3	5.5	4	3.5
CCQ, mental, after EMDR	1 *	3.5*	3*	2.5*
CRQ, emotional, before EMDR	3.8	2.3	2.7	3.7
CRQ, emotional, after EMDR	5.8*	5.3*	2.7	5.2*
CRQ, mastery, before EMDR	2.3	2.5	2.5	3
CRQ, mastery,after EMDR	5.3*	5.8*	4.3*	4.3*

For SQ subset anxiety, values above 11 suggest high levels of anxiety. For HADS-A, values above 8 suggest high levels of anxiety. For CCQ, lower scores indicate a more favorable health status. For CRQ, higher scores indicate a more favorable health status

*Clinically relevant change

### Case 1

Patient E., female, aged 63, was referred for EMDR after hospitalization for an AECOPD because of fear for breathlessness. During her intake session, she described a deep fear of suffocation, panic attacks related to her COPD, and fear to go out of the house without taking a benzodiazepine. She had been claustrophobic since she had been rolled into a carpet as a four-years-old by other children and could not get out. After her intake session with the psychiatrist, she was diagnosed with agoraphobia and panic disorder. Previously, she had been diagnosed with carcinophobia. She used oxazepam 10 mg three times daily. She did not drink alcohol.

EMDR was provided, targeting memories and disaster scenarios. The memories were: being breathless, breathlessness in the shower, being rolled into a carpet. The mental images or disaster scenarios were: dying by suffocation, the idea of having cancer in her throat, sitting on a chair unable to move, hearing the doorbell ring unexpectedly. She had five sessions (between December ‘19 and February ‘20).

On CCQ, measured before and after EMDR, she showed no (significant) change on symptoms or functioning, but a large improvement (2 points) in mental status. Unfortunately, no HADS was measured in this patient. On CRQ, subscales dyspnea and fatigue remained unchanged but subsets emotion and mastery both showed a large improvement of 2 points. Regarding SQ48, there was a large improvement in anxiety (5 points) and some improvement in vitality. Other subsets remained unchanged.

### Case 2

Patient B., male, aged 69 years, asked for referral to undergo EMDR, because of fear of suffocation. He avoided activity for fear of breathlessness and had constant thoughts of suffocating. In his life, several important events happened that concerned breathing: there had been two events of near drowning when he was young; he had caused a fire in his garage (before he had COPD), and he had had a severe breathlessness crisis in his car due to COPD. Of those events, he deemed the breathlessness crisis in his car essential to his anxiety. Previously, he had been diagnosed with depression, agoraphobia and panic disorder. He took oxazepam (5 mg) PRN. He did not drink alcohol.

EMDR targeted memories and disaster scenarios. The memories were: breathlessness crisis in the car and becoming breathless while walking to car. Disaster scenarios were: dying while in the shower, becoming unable to drive his car, panic attack in the presence of a stranger, and becoming breathless in a crowded cinema. He had four EMDR sessions (between April and May ‘20).

On CCQ, measured before and after EMDR, he showed an improvement on all subsets (0.8 points on symptoms, 0.5 points on functioning and 2 points on mental status). He had high scores on HADS-A before treatment, which declined significantly with 4 points but not below 9. Regarding SQ48, he showed some improvement on most subsets, except vitality.

### Case 3

Patient P. male, aged 79, asked for referral for EMDR because he had heard that it could be effective. He suffered from fear of suffocation and described overwhelming anxiety when he thought of breathlessness and of dying. He had no specific breathlessness episodes in his life, other than general breathlessness related to COPD. He used no psychotropic drugs and drank two units of alcohol per day.

EMDR was provided, targeting both memories and mental images or disaster scenarios. The memories were: Breathlessness during an exacerbation of COPD and the death of his father. Disaster scenarios: being strangled; dying, choking, while the hospital staff cannot help him. He had four EMDR sessions (between February and June ‘20). Part of the EMDR was done through video calling because of the COVID-19 pandemic.

On CCQ, measured before and after EMDR, he showed a deterioration in symptoms (0.5 points), but a significant improvement in mental status (1 point); functioning remained unchanged. HADS-A showed a great improvement (6 points) but stayed above 8. On CRQ, subscales dyspnea and emotion remained unchanged, fatigue deteriorated (1.5 points), but mastery showed a large improvement (1.75 points). Regarding SQ48, he showed only a small improvement in anxiety and social phobia; the domains vitality and cognitive complaints deteriorated somewhat. Other subsets remained unchanged.

### Case 4

Patient H., female, aged 65, had been referred for EMDR before because of fear of suffocation. During the intake, she gave a detailed description of the onset of AECOPD a year before the referral. During this breathlessness crisis, she was unable to wake up her husband. She experienced fear of suffocation, flashbacks and fear to leave the house. She had no previous psychiatric history but at the start of EMDR, PTSS was diagnosed. She used one unit alcohol daily, took morphine 10 mg SR twice daily for breathlessness, and had recently started taking oxazepam 10 mg PRN.

Because of visual impairment, EMDR was provided with two vibration devices (buzzers). The target memories were the described breathlessness crisis, and a breathlessness episode she had experienced while taking a shower. She had three EMDR sessions (between March and May ‘21).

On CCQ, measured before and after EMDR, she showed a deterioration in symptoms, but a significant improvement in mental status and functioning (both 1 point). HADS-A declined with 5 points to a normal value. On CRQ, subscale dyspnea could not be assessed because the patient did not fill in that part of the questionnaire. She improved markedly on emotion (1.5 points), fatigue (2 points) and mastery (1.3 points). Regarding SQ48, she showed a great improvement in anxiety (12 points), agoraphobia (6 points, score normalized to 0), social phobia (4 points, score normalized to 0), somatic complaints (13 points) and depression (15 points, score normalized to 2). Nonetheless, she deteriorated on the domain vitality with 7 points.

## Results qualitative interviews

The initial number of 101 codes could be merged into 18 categories, which were grouped into four themes: trauma, panic and anxiety, breathlessness and effect of EMDR.

### Trauma

All four patients described psychotraumatic episodes related to breathing. For patient 1, the main event was being rolled into a carpet by other children when she was very young. She described how, since then, she was afraid of confined spaces and did not tolerate anything on her face. In her opinion, this event was more significant than the breathlessness she experienced because of her COPD.

The psychotraumatic breathing episodes described by the other three patients were mostly COPD related. Besides, patient 2 described two episodes of near drowning, as well as being in a fire in his garage, but felt that the breathlessness related to his COPD had much more impact.

The fire and the drowning were not that bad. But the stepping into the car… (….) I got a panic attack that made me rigid. (…) It was very oppressive and very frightening.

### Panic and anxiety

All four patients described high levels of anxiety related to breathing. One patient described how breathlessness would lead to shaking, suggesting a linkage with fear.

You feel how you can’t breathe, or hardly. Or you start shaking. When I walk during physio, walk three times, I start shaking.

(Patient 4.)

They described how certain events triggered anxiety: wind (patient 1), having a shower (patient 1, patient 2, patient 4), having to go to the toilet (patient 1), being alone (patient 2), going out (patient 2, patient 3). Moreover, all four patients described fear of suffocation.

The idea of sudden breathlessness in a public space provoked anxiety in patient 2. Patient 1 described loss of control:

I get so breathless, it becomes a panic attack. Because you just can’t control it anymore.

(Patient 1)

Patient 2 and 3 described fear of dying.

Like a very nasty death struggle due to the breathlessness, a very painful affair. I could picture that in my mind.

(Patient 3)

Patient 2 and 3 described an increase of anxiety after something witnessed in other patients (on the first aid or on the ward).

He was wheeled in and started arranging his euthanasia, while I was lying next to him. It got to me, it really did. It came really close. I got this panicky feeling, feeling of suffocating and not being able to come out of it. Some sort of stranglehold, I got.

(Patient 3)

Of note, both patients 1 and 4 described being claustrophobic, both were afraid of confined spaces and did not tolerate anything on their face.

Yes, just don’t let them touch my face or my head. And I am always on the outside of doors, and the doors must be open, you know. (Patient 4).

### Breathlessness

Patient 1, 2 and 4 described frequent unpredictable breathlessness crises that were not triggered by exercise or an exacerbation. Patient 4 described how the memory of a sudden episode of breathlessness when she was sitting on a specific chair in the house, would come back when she sat in the same chair. Patient 2 described how an episode of breathlessness reminded him of the severe crisis he had while sitting in a car. Patient 2 described how breathlessness could be induced by thoughts:

When I go to bed at night, I think, oh I didn’t get breathless, and then that’s when I get it.” (Patient 2).

### Effect of EMDR

#### On anxiety

All four patients described being less anxious after EMDR. In all four patients, fear of suffocation had disappeared or greatly diminished. Patient 2 described that, since EMDR, he no longer feared a sudden breathlessness episode in a public space, and felt more confident to go out.

#### On breathlessness

Concerning breathlessness, all four patients made a distinction between ‘regular’ breathlessness and breathlessness with anxiety. They all stated that the breathlessness in itself did not change, but the intertwined anxiety diminished.

Because at a certain point, I was breathless all day, also because of the panic. Now I am breathless, but only when I do something. The normal breathlessness that comes with the COPD. That I can handle, by the way. Not always, but most of the time. Panic breathless you just can’t handle. You try, but you can’t. (Patient 1).

The breathlessness in itself did not change. Just the link with anxiety disappeared. The breathlessness is there, that won’t disappear with EMDR. I still have that. When I am breathless now, I don’t have the fear that’s linked to it, like o my god here comes that stranglehold. (Patient 3).

The breathlessness is still there, but I deal with it differently, I tolerate it better. It’s a different feeling. A lot less fear. Less panic. (Patient 2).

Usually when I have to go to the bathroom, I think o I have to go, o God o God. If only I don’t get frightened, I think. (…) But at a certain point I thought, I have been to the bathroom three times without realizing it thinking or about it. O it helps. O it helps!! I called my sister right away, it helps it helps. I have been to the bathroom three times! (Patient 1)

#### On coping with COPD

Patient 2 described how he was not bothered anymore about what other people think.

Those things I say, I am going to do things in stages, and I do not care a rap if anybody sits next to me and I get breathless. They know it, they will have to accept it. I don’t have to think about them, they should be thinking about me.

Patient 1, 2 and 4 described how they had changed their daily routines since EMDR, suggesting a better coping style.

For example, I have done the laundry and I need to bend over to take it out. Then I need to hang it. You can do it in stages, you can and you should. And I thought, if I just do it all at once, I get it over with. But I don’t do that anymore, not since the EMDR. (Patient 1.)

#### Recommendation for other patients

When asked whether they would recommend EMDR for other COPD patients, all four subjects answered positively. Patients 1 and 3 had already advised a fellow patient to try EMDR. Patient 2 noted that EMDR would not benefit all patients with COPD, only those who experience panic. Patient 4 stated that it was helpful for anxiety, but not for breathlessness.

#### Side-effects

Patient 1 mentioned some fatigue after EMDR. No other side-effects of the intervention (such as increased breathlessness during EMDR) were mentioned by the study subjects.

## Discussion

The present case series suggests a potential benefit of EMDR in a subset of COPD patients, who have experienced potential psychotraumatic episodes of breathlessness. In the qualitative interviews, patients illustrated how such memories enhance and intensify the experience of breathlessness symptoms. Indeed, breathlessness could be triggered by a situation where a previous breathlessness crisis had taken place, or even by thoughts. All patients reported high levels of anxiety and described how breathlessness and anxiety were intertwined.

After three to five EMDR sessions, all four patients had a notable effect on the mastery domain of CRQ. EMDR decreased levels of anxiety (according to the interviews, SQ subset anxiety, and the HADS-A that was taken in three out of four patients). Interestingly, they all distinguished ‘normal breathlessness’ from ‘panic breathlessness’ after EMDR. They all stated that normal breathlessness wasn’t altered by this intervention, which is illustrated by the fact that CCQ, subset symptoms did not improve in three out of four patients. However, the ‘panic breathlessness’ improved greatly. This supports our hypothesis that previous psychotraumatic breathlessness episodes play an important role in symptom experience, and maybe also in quality of life. Three out of the four patients were able to describe psychotraumatic breathlessness crises in detail (case 1, 2 and 4). According to the questionnaires, they had more benefit of EMDR than patient 3, who suffered from fear of suffocation but did not have a specific memory of a breathlessness crisis. This is in accordance with the leading explanation for the mechanism behind EMDR, in which an aversive memory is pivotal [17].

Interestingly, three out of four patients described how they had better ways to cope with their breathlessness after EMDR. This might also be a result of the psychoeducation that was given prior to EMDR referral (including the explanation that patients with COPD do not literally suffocate, because the airways stay open).

EMDR is a safe, well tolerated, fast and low-cost intervention that has rapidly been gaining popularity in the last decades [15]. The fact that all four patients stated that they recommended the intervention for other COPD patients is telling. However, to assess its possible place in the management of breathlessness, a randomized controlled trial is necessary in which EMDR is compared to psychoeducation. Eligible patients should have severe breathlessness due to a chronic illness with episodic breathlessness (such as COPD or heart failure), high levels of anxiety and a vivid memory of a breathlessness crisis.

## Strengths and weaknesses

To our knowledge, this is the first study exploring the possible effect of EMDR in breathlessness in COPD. The combination of semi-structured interviews and validated questionnaires has been helpful to explore this promising intervention and to strengthen our hypothesis.

The low number of included patients is a weakness. The number of four interviews is too low to reach saturation; indeed, every interview yielded new codes. We have tried to find more patients for our interviews via other therapists but did not succeed. Therefore, a prospective study should ideally have a mixed-methods design to further explore patient experience of this intervention. Furthermore, it is important to measure long-term effects, which has not been done in this case series.

## Conclusion

According to this case series, EMDR is a promising intervention in patients with refractory breathlessness in combination with anxiety, who have vivid memories of breathlessness episodes. Our findings validate the design of a randomized controlled clinical trial on this subject.

## Supplementary Information

**Additional file 1**. PRISMA flow diagram of literature search.

## Data Availability

The data that support the findings of this study are not publicly available due to reasons of privacy, but are available from the corresponding author upon reasonable request.

## Abbreviations

AECOPD: Acute exacerbation of chronic obstructive pulmonary disease
CCQ: Clinical COPD Questionnaire
COPD: Chronic obstructive pulmonary disease
CRQ: Chronic Respiratory Disease Questionnaire
EMDR: Eye movement desensitization and reprocessing
FEV1: Forced expiratory volume in one second
HADS-A: Hospital Anxiety and Depression Scale-anxiety
HADS-D: Hospital Anxiety and Depression Scale-depression
mMRC: Modified Medical Research Counsil
PTSD: Post-traumatic stress disorder
SQ48: 48-item Symptom Questionnaire
