# Supplementary material for: “When I am breathless now, I don’t have the fear that’s linked to it”: a case series on the potential of EMDR to break the dyspnea-anxiety cycle in COPD
Source: BMC Pulm Med. 2022 Dec 1;22:456. doi: 10.1186/s12890-022-02250-1 (PMC9713157; doi:10.1186/s12890-022-02250-1)
Supplement: Supplementary file 1 — Additional file 1. PRISMA flow diagram of literature search. [file 12890_2022_2250_MOESM1_ESM.docx]

**Identification of studies via databases and registers**

Records removed *before screening*:

Duplicate records removed (n = 1)

Records marked as ineligible by automation tools (n = 0)

Records removed for other reasons (n = 0)

Records identified from*:

Databases;

1. PubMed n = 3
2. PsycInfo: n=1

Web search engines (Google Scholar) (n = 625)

**Identification**

Records screened

(n = 200)

Records excluded**

(n = 199)

Reports sought for retrieval

(n = 3)

Reports not retrieved

(n = 0)

**Screening**

Reports assessed for eligibility

(n = 3)

Reports excluded:

Reason 1: does not concern breathlessness in COPD (n =3 )

Studies included in review

(n = 0

Reports of included studies

(n = 0

**Included**

Search terms:

Pubmed: ((("eye movement desensitization reprocessing"[MeSH Terms] OR ("eye"[All Fields] AND "movement"[All Fields] AND "desensitization"[All Fields] AND "reprocessing"[All Fields]) OR "eye movement desensitization reprocessing"[All Fields] OR "emdr"[All Fields]) AND (((("pulmonary disease, chronic obstructive"[MeSH Terms] OR ("pulmonary"[All Fields] AND "disease"[All Fields] AND "chronic"[All Fields] AND "obstructive"[All Fields]) OR "chronic obstructive pulmonary disease"[All Fields] OR "copd"[All Fields])) OR ("dyspnoea"[All Fields] OR "dyspnea"[MeSH Terms] OR "dyspnea"[All Fields])) OR ("asphyxia"[MeSH Terms] OR "asphyxia"[All Fields] OR "suffocation"[All Fields] OR "suffocating"[All Fields])

PsycInfo: (COPD/ OR Dyspnea.mp) AND EMDR/

Google Scholar: EMDR dyspnea OR breathlessness OR COPD OR fear of suffocation

Our literature search retrieved no publications on EMDR in COPD patients. We identified two case reports on the effect of EMDR on breathlessness in other conditions. The first case report described a 16-year-old girl who suffered from asthma-specific fear (1). After EMDR and cognitive behavioral therapy, her asthma symptoms were reduced. The second case report described the positive result of EMDR in a patient with amyotrophic lateral sclerosis on PTSD symptomatology following suffocation due to a blocked tracheal cannula (2).

*(1) Verkleij M., Maric M., Colland V., Nagelkerke A. F., Geenen R. (2017). Cognitive-behavioral therapy and eye movement desensitization and reprocessing in an adolescent with difficult-to-control asthma. Pediatric Allergy, Immunology, and Pulmonology. Jun 2017:103-112*

*(2) Oudman E, Baert J. Eye movement desensitization and reprocessing (EMDR) and mediative behavioral therapy for the treatment of suffocation related post-traumatic stress disorder (PTSD) in amyotrophic lateral sclerosis (ALS): A case report. Palliat Support Care. 2022 May 11;1-3.*
